# Investigation of the GnRH antagonist degarelix isomerization in biological matrices

**DOI:** 10.1002/prp2.1117

**Published:** 2023-07-16

**Authors:** Lucia Ferrazzano, Alessandra Tolomelli, Ivan Guryanov, Marco Macis, Ulrich Abel, Antonio Ricci, Walter Cabri

**Affiliations:** ^1^ Department of Chemistry “Giacomo Ciamician”, Alma Mater Studiorum University of Bologna Bologna Italy; ^2^ Fresenius Kabi iPSUM Srl Villadose (RO) Italy; ^3^ Institute of Chemistry St. Petersburg State University St. Petersburg Russia; ^4^ Fresenius Kabi Deutschland GmbH Bad Homburg Germany

**Keywords:** degarelix, dihydroorotate, hydantoin, metabolism, peptide

## Abstract

One of the main objectives of peptide drug design is the improvement of peptide pharmacokinetics with maintaining biological activity, which can be achieved by the complex modifications of the primary structure of the peptides. However, these changes often lead to the formation of peculiar impurities in the peptide drugs and their metabolites, which require the development of advanced analytical methods to properly assess their content. Here, we investigated the degradation of the potent long‐acting GnRH antagonist degarelix in various biologic media by the tailor‐made HPLC method, which allows precise determination of 5‐Aph(Hyd)‐degarelix isomer, an impurity found in the degarelix active pharmaceutical ingredient (API) during its manufacturing. Unexpectedly, we discovered a rapid and irreversible conversion of degarelix API into the corresponding hydantoin isomer in serum, suggesting that this impurity can be also a potential drug metabolite in vivo. This finding underlines the importance of the development of more accurate and performing analytical techniques to correctly characterize the chemical composition of the manufactured drugs and their behavior under physiological conditions.

## Graphical abstract

We investigated the degradation of the GnRH antagonist Degarelix in various biologic media by the tailor‐made HPLC method, which allows precise determination of 5‐Aph(Hyd)‐Degarelix isomer, and we discovered a rapid and irreversible conversion of Degarelix API into the corresponding hydantoin isomer in serum, suggesting that it can be a potential drug metabolite in vivo. This finding can improve the understanding of the metabolic pathways of Degarelix.

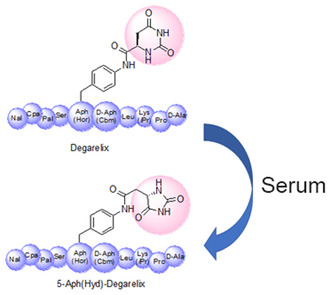

**Abbreviations**
API: active pharmaceutical ingredient
GnRH: gonadotropin‐releasing hormone
Hyd: hydantoin
HSA: human serum albumin

## 1 INTRODUCTION

Peptide therapeutics are among the most promising classes of medicines due to their high affinity and selectivity to biological targets.^1^ However, the use of natural peptides as drugs is limited because of their poor pharmacokinetics caused by rapid metabolic degradation and fast clearance.^2^ Various methods to improve ADME (Absorption, Distribution, Metabolism, and Excretion) properties of the peptides have been proposed, including hydrogen bond surrogates, stapled structures, the introduction of rigid backbone linkers, and the addition of non‐proteinogenic amino acids or modified moieties.^3^, ^4^, ^5^ A remarkable example of the last approach is the gonadotropin‐releasing hormone (GnRH) derivatives used for prostate cancer treatment.

**FIGURE 1:**
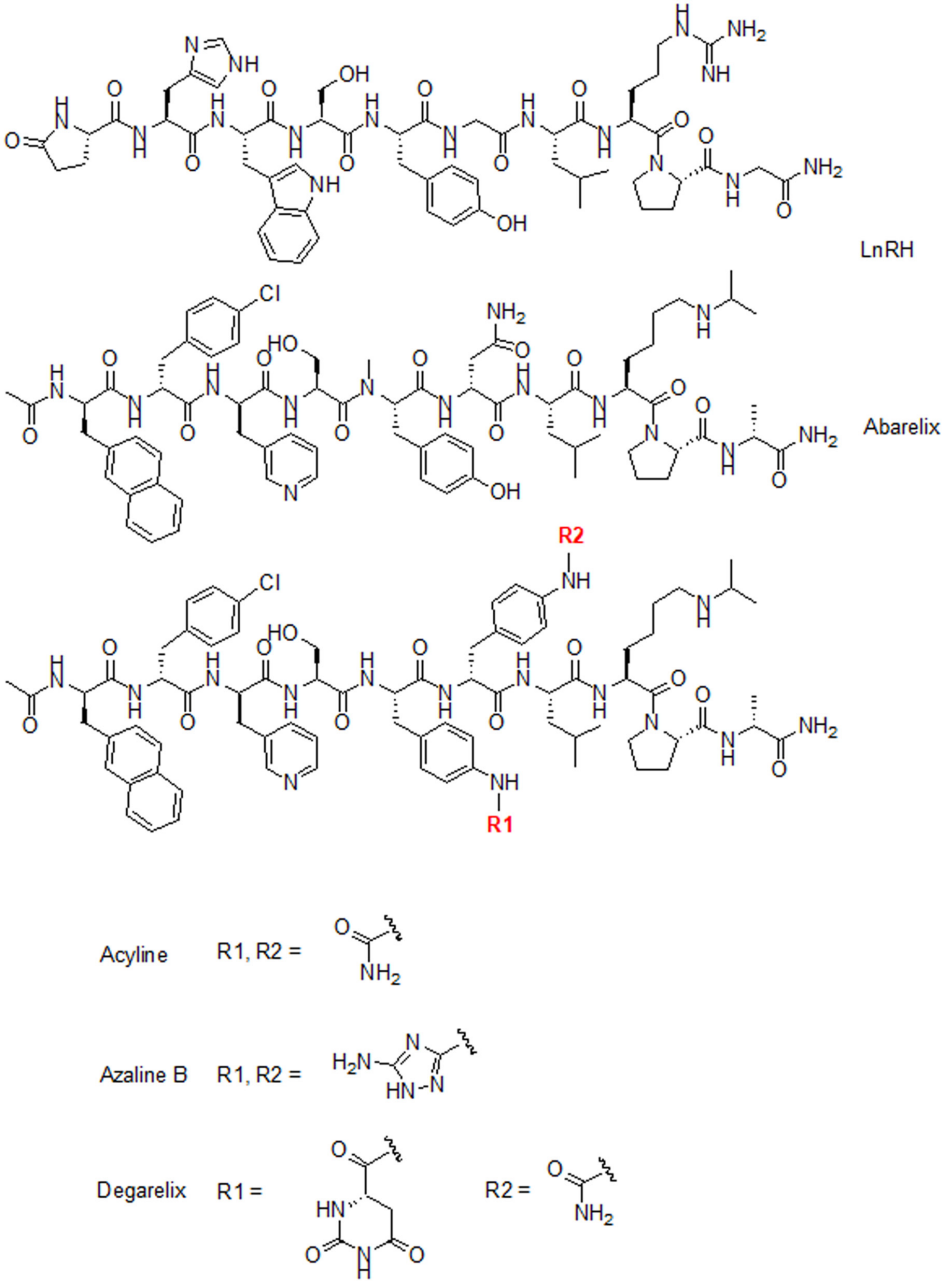
Structures of gonadotropin‐releasing hormone, abarelix, acyline, azaline B, and degarelix.

Due to the sensitivity of the advanced forms of prostate cancer to the serum testosterone level, androgen deprivation with GnRH antagonists became an efficient anticancer therapy.^6^, ^7^, ^8^ The GnRH antagonists currently used in clinical practice for prostate cancer treatment are derivatives of the natural decapeptide, where seven out of the ten residues are substituted with non‐proteinogenic amino acids (Figure 1).

**FIGURE 2:**
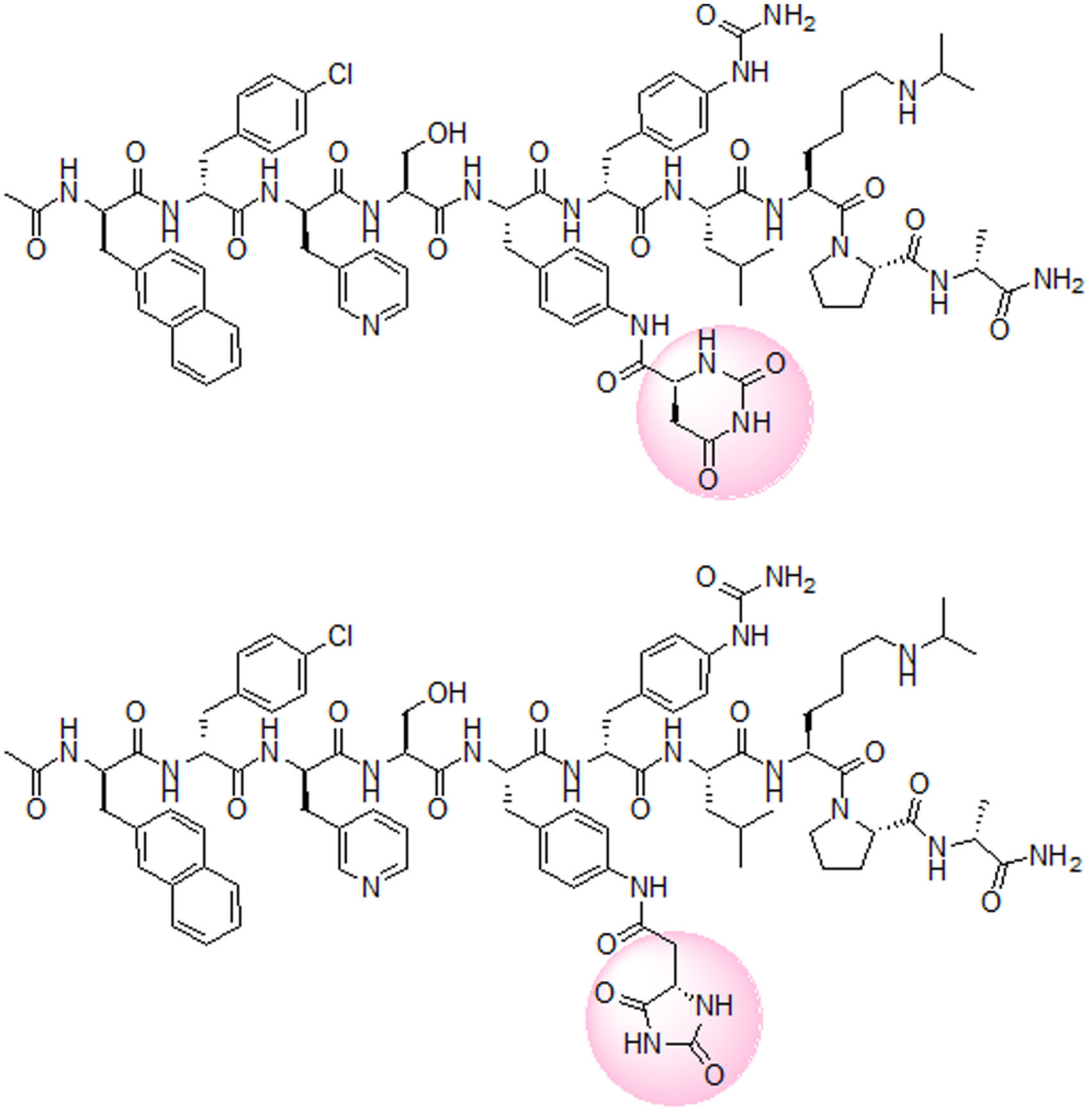
Structures of degarelix (top) and 5‐Aph(Hyd)‐degarelix (bottom).

These substitutions strongly influenced the physicochemical and biological properties of the peptides. Starting from abarelix, the first GnRH antagonist approved by FDA, which showed noticeable allergic effects and was withdrawn lately from the US market, modifications of the natural GnRH led to the design of potent and long‐acting inhibitors, such as azaline B and acyline.^9^, ^10^, ^11^, ^12^ Further modifications of the azaline B molecule resulted in the development of third generation GnRH antagonist degarelix with an improved pharmacological profile (Figure 2).^13^, ^14^, ^15^, ^16^

**FIGURE 3:**
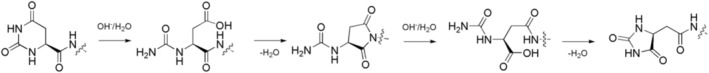
Rearrangement of the Hor residue in the presence of bases.

After its approval by FDA in 2008, degarelix became the most widely used GnRH antagonist in patients with advanced prostate cancer.^17^ The advantages of degarelix are its high affinity to the GnRH receptor, increased hydrophilicity, and decreased propensity to form gels. As a result, degarelix has better bioavailability than the previous GnRH antagonists. These improved characteristics are related to the presence of additional 4‐[(aminocarbonyl)amino]‐phenylalanine [Aph(Cbm)] and 4‐[(dihydroorotyl)amino]‐phenylalanine [Aph(Hor)] moieties, which afford multiple hydrogen‐bonding sites and permit self‐association with the formation of “amyloid” type fibrils, which dissolve over a long period (amyloid *t*_1/2_ = 15 days).^18^, ^19^ However, the insertion of these non‐proteinogenic amino acids poses several challenges for the manufacturing of degarelix. The Aph(Hor) moiety is known to be rapidly hydrolyzed under basic conditions to the *N*‐carbamoyl aspartyl intermediate with further rearrangement to the five‐membered hydantoin isomer (Figure 3).^20^, ^21^ Since the current standard manufacturing routes for peptide assembly rely on the solid‐phase peptide synthesis (SPPS) approach, which requires the use of a base (usually piperidine) to achieve the deprotection of the FMOC group, the abovementioned rearrangement has to be considered unavoidable and the related impurity 5‐Aph(Hyd)‐degarelix (where Hyd = hydantoin) can be constantly present in the final degarelix active pharmaceutical ingredient (API).^22^ For this reason, the potential formation of this impurity under physiological conditions plays a crucial role in the understanding of its potential toxicity profile. Indeed, degarelix binding to human serum albumin (HSA) is shown to be very high (76.3%) and the presence of many basic residues (58 lysines and 27 histidines) in the protein sequence and the binding sites could favor the isomerization process.^23^, ^24^, ^25^ The detection and identification of metabolites are generally performed using HPLC‐MS or Ultra‐Performance Liquid Chromatography (UPLC) methods and it becomes very simple when the metabolite has a different molecular weight. On the other hand, 5‐Aph(Hyd)‐degarelix impurity has the exact molecular weight of the parent drug and, unfortunately, its proper quantification is complicated because of the high similarity of its structural and physio‐chemical properties to those of the parent peptide (Figure 2). Thus, the development of a tailored analytical method became necessary to properly evaluate its content in the degarelix API.

Previously, to evaluate the hydantoin impurity content in degarelix as an active pharmaceutical ingredient we developed an improved analytical method, which allows an excellent separation of degarelix and 5‐Aph(Hyd)‐degarelix,^26^ and we applied it for the study of the stability of the peptide in various biological environments.

## 2 MATERIALS AND METHODS

Analytical grade reagents and solvents were purchased in Merck and used without further purification. Degarelix API and 5‐Aph(Hyd)‐degarelix were prepared in Xingyin Pharmaceutical. Human liver microsomes (pool of 50 male and female donors) were provided by Prolytic GmbH. Human male AB plasma (USA origin, sterile filtered) was obtained from Merck.

### 2.1 HPLC chromatography

*Analytical method A*: Agilent 1260 instrument; Phenomenex Gemini NX‐C18 150 × 4.6 mm, 3.0 μm column; column temperature 25°C; Eluent—10 mM NH_4_OAc (pH 9.5)/ACN 6:4; isocratic elution (run time 20 min).

*Analytical method B*: Agilent 1260 instrument; Waters Acquity BEH C18 150 × 3.0 mm, 1.7 μm column; column temperature 45°C; Eluent A—H_2_O/ACN 9:1 + 0.1% methanesulfonic acid; Eluent B—H_2_O/ACN 1:9 + 0.1% methanesulfonic acid; gradient elution: 0.00 min—20% of eluent B, 25.00 min—25% of eluent B; 40.00 min—60% of eluent B; 45.00 min—60% of eluent B; 46.00 min—20% of eluent B; 53.00 min—20% of eluent B.

### 2.2 Mass spectrometry analysis

Mass spectra were acquired on API 4000 spectrometer operating in the positive mode.

### 2.3 Linearity investigation of the Analytical method A

The calibration standards were prepared in the same media, which were used for the stability tests. The calibration curves were in the range of 20–2000 ng/mL for degarelix and 5‐Aph(Hyd)‐degarelix and showed acceptable linearity over the calibration range for all media. The linearity of the Analytical method A was tested for the mixture of 5‐Aph(Hyd)‐degarelix and degarelix (2 μg/mL) for the concentration of the 5‐Aph(Hyd)‐degarelix 0.01 μg/mL, 0.02 μg/mL, and 0.1 μg/mL. The stability of degarelix in the conditions of the Analytical method A was tested by dissolving it in the mobile phase at the concentration of 2 μg/mL and incubating for 2 h at 25°C.

### 2.4 Stability investigations in different solutions

Degarelix or 5‐Hyd‐degarelix test solutions (10 μL of 180 μg/mL degarelix or 5‐Aph(Hyd)‐degarelix in 20% acetonitrile) were each mixed with 890 μL of human plasma, Dulbecco buffer (5 mM NaH_2_PO_4_, 20 mM Na_2_HPO_4_, 5 mM KCl, 120 mM NaCl, adjusted to pH 7.4 with NaOH), 4.5% human serum albumin solution (HSA solution), and human serum albumin solution with 0.21 mg/mL warfarin: 3960 μL HSA solution +40 μL warfarin stock solution (21 mg warfarin in 1 mL water)to a final concentration of 2000 ng/mL of each analyte and 0.2% of acetonitrile. Three aliquots of 50 μL were prepared for different time intervals (0, 8, 24, 32, and 48 h) and incubated at 37°C or 5°C. pH of the solutions was controlled during the experiments and did not show any change (pH = 7.8 for human serum and 7.4 for Dulbecco buffer). The samples were then diluted with 10 μL of the solution of 0.1% of formic acid in 20% acetonitrile and 70 μL of acetonitrile. The solutions were shaken and centrifuged. To 50 μL of the supernatant, 20 μL of water and 0.2% of formic acid were added and the solution was used for HPLC analysis. All the experiments were repeated three times.

### 2.5 Stability investigations in human liver microsomes

The human liver microsome suspensions (protein concentration 20 mg/mL) were thawed for approximately 2 min in a water bath at RT and further kept on ice. After shaking the solution for 5–10 s, the required amount of microsomes was removed and the remaining sample was immediately re‐frozen at approximately −80°C.

To the test samples containing human liver microsomes (25 μL with 20 mg microsomal protein/mL and 345 μL of PBS), 5 μL of degarelix test solution (200 μg/mL in 10% acetonitrile) was added. The samples were incubated at 37°C for 0 min (reference sample), and at different time intervals with or without 125 μL of 0.5 mg/mL NADPH. In the case of the absence of NADPH 125 μL of the solution of 2 mg/mL glucose‐6‐phosphate and 0.45 μg/mL glucose‐6‐phosphate dehydrogenase in 2% NaHCO_3_ were added. Following the addition of 500 μL acetonitrile, the samples were centrifuged and 50 μL of the supernatant of each sample were diluted with 20 μL of 0.2% formic acid in water and used for HPLC analysis.

### 2.6 Data evaluation

The ANALYST software was used to integrate all peaks automatically by use of the IntelliQuan algorithm and to calculate the calibration curves of degarelix and 5‐Aph(Hyd)‐degarelix by linear regression with a weighting of 1/*x* ^2^. The parameters for peak integrations were adapted for all samples in each analytical batch. The ANALYST software was used to calculate the concentrations of the test and QC samples based on the corresponding calibration curves. The standard deviation was calculated as SD = $\sqrt{\frac{n\sum x^{2}-{\left(\sum x\right)}^{2}}{n\left(n-1\right).}}$

## 3 RESULTS

**FIGURE 4:**
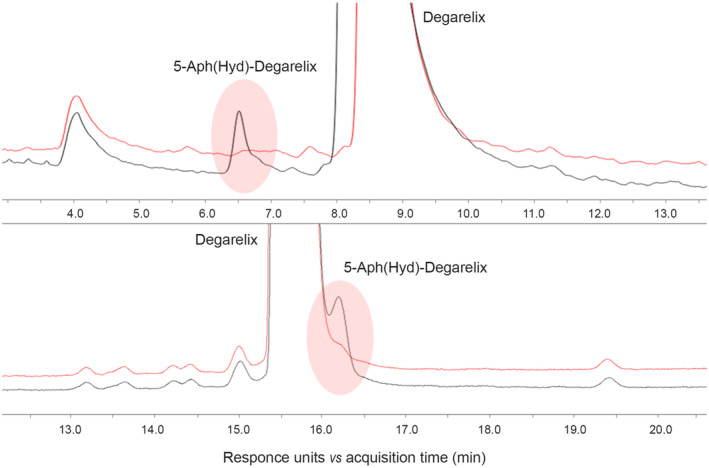
Comparison of degarelix API (red) and degarelix API with the addition of 0.5% (*w/w*) of the 5‐Aph(Hyd)‐degarelix (black) using basic eluent (Analytical method A, top) and acidic eluent (Analytical method B, bottom) (see Materials and Methods for details).

Several experiments performed in our laboratory demonstrated that the proper separation of the 5‐Aph(Hyd)‐degarelix isomer from degarelix is impossible with standard chromatographic methods with acidic mobile phases even when the UPLC method is applied. On the contrary, the application of a basic mobile phase allows an excellent separation of the two peaks, allowing the adequate measurement of the content of this impurity in the degarelix API (Figure 4, Table 1).^26^ The basic conditions of this analytical method do not contribute to the formation of the 5‐Aph(Hyd)‐degarelix isomer, as it was confirmed in the experiments performed during the analytical method validation activities. Only after 2 h 0.19% of 5‐Aph(Hyd)‐degarelix was found when degarelix was dissolved in the mobile phase, which is sufficient to carry out HPLC analysis.

**TABLE 1:** Linearity investigation of the Analytical method A (see Materials and Methods for details).

Degarelix concentration, μg/mL	Theoretical content	Theoretical concentration	Experimental content
	5‐Aph(Hyd)‐degarelix, %	5‐Aph(Hyd)‐degarelix, μg/mL	5‐Aph(Hyd)‐degarelix, %
2.0	0.5	0.01	0.45
	1.0	0.02	0.99
	5.0	0.1	5.58

**FIGURE 5:**
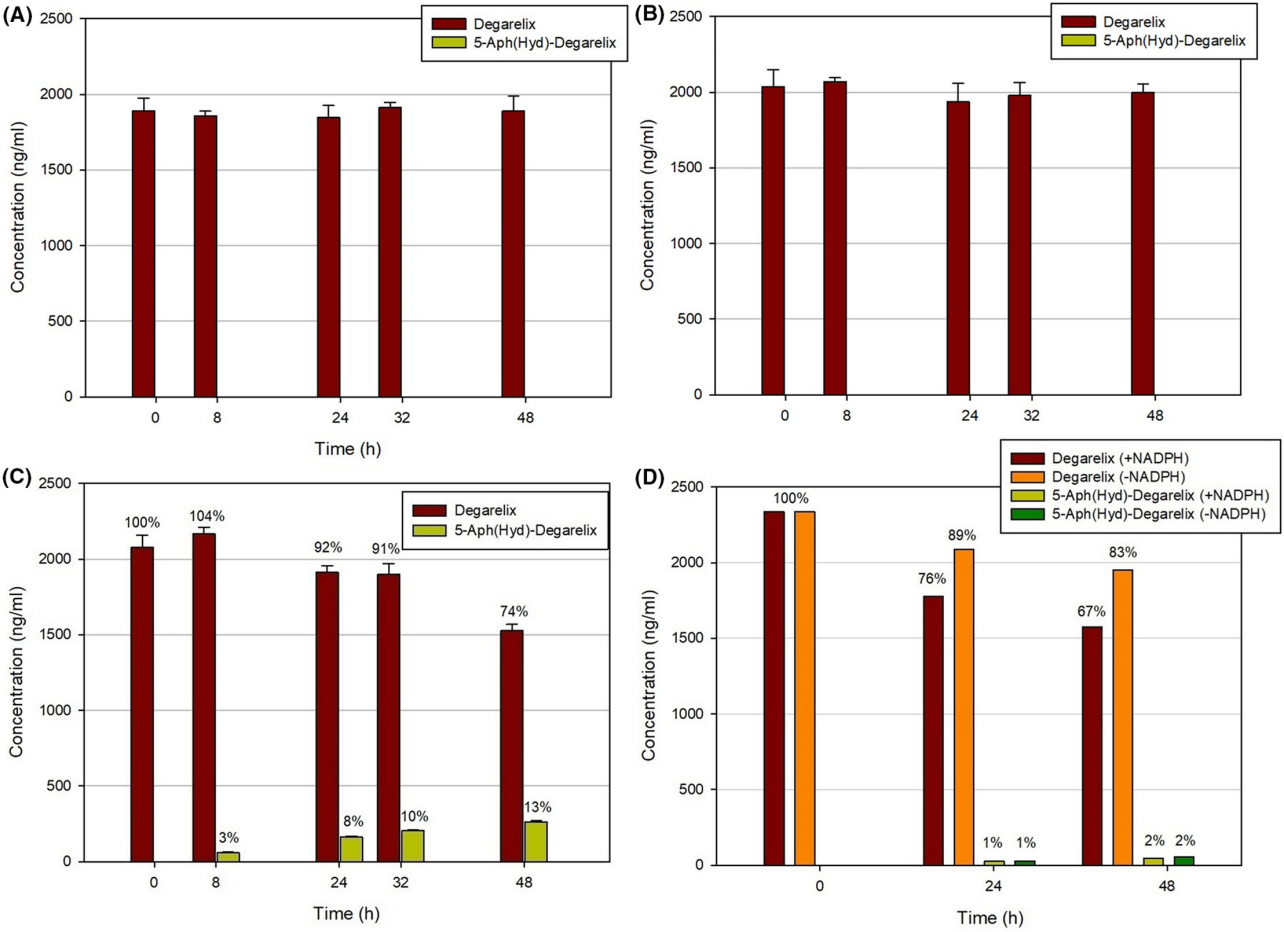
Stability of degarelix in human serum albumin (has) solution without warfarin (A), in HSA solution with warfarin (B), human plasma (C), and in human liver microsomes (D).

**FIGURE 6:**
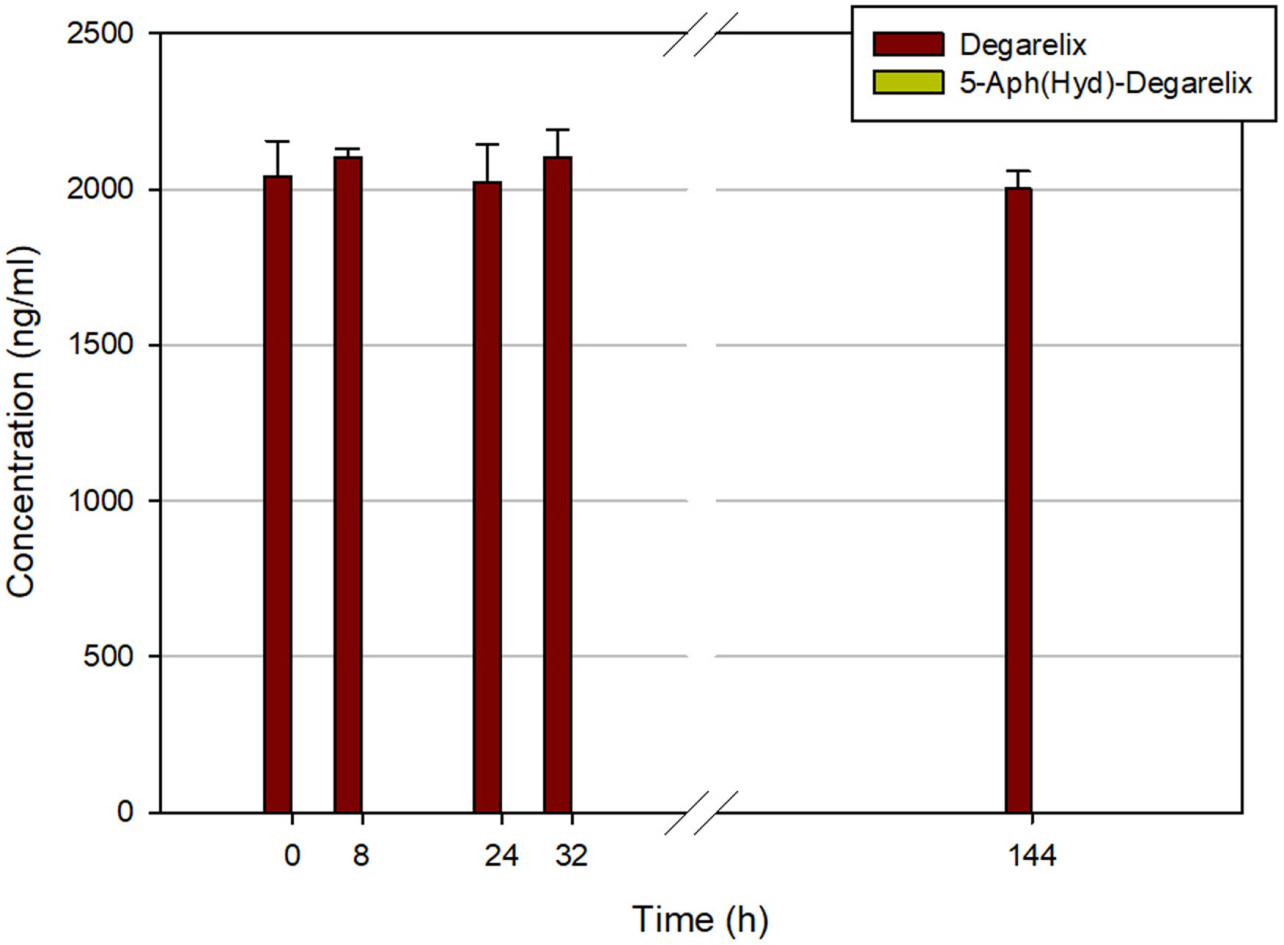
Stability of degarelix in human plasma at 5°C.

The study of the degradation was performed in human plasma, human serum albumin solution (as a surrogate of serum matrix), and HSA solution with the addition of the binding blocker warfarin in equimolar concentration to albumin.^27^ A Dulbecco buffer at pH 7.4 was taken as a reference, where the degarelix was shown to be stable over a prolonged period. All the solutions were incubated at 37°C and the samples were analyzed by HPLC‐MS/MS at different time intervals using Analytical method A.^26^ After 48 h of incubation in human plasma, the 5‐Aph(Hyd)‐degarelix formation was detected to a level up to 13% in the overall degradation of 25%–28% of the initial amount of the peptide (Figure 5). On the contrary, no hydantoin isomer was found in the case of incubation in human plasma at 5°C (Figure 6). No 5‐Aph(Hyd)‐degarelix was observed in human serum albumin solutions with or without warfarin. Furthermore, the same trend was observed when albumin solution was added with amino acids or ions normally present in the human plasma (data not shown).^28^ The rate of formation of the 5‐Aph(Hyd)‐degarelix in human liver microsomes was much slower and it barely depended on the presence of NADPH, confirming that the absence of the involvement of the cytochrome P450 system in its formation (Figure 5D). However, the concentration of the degarelix in solution was markedly reduced after 48 h (about 33% in the presence of NADPH), which indicates a metabolic pathway different from the dihydroorotate‐hydantoin isomerization.

**FIGURE 7:**
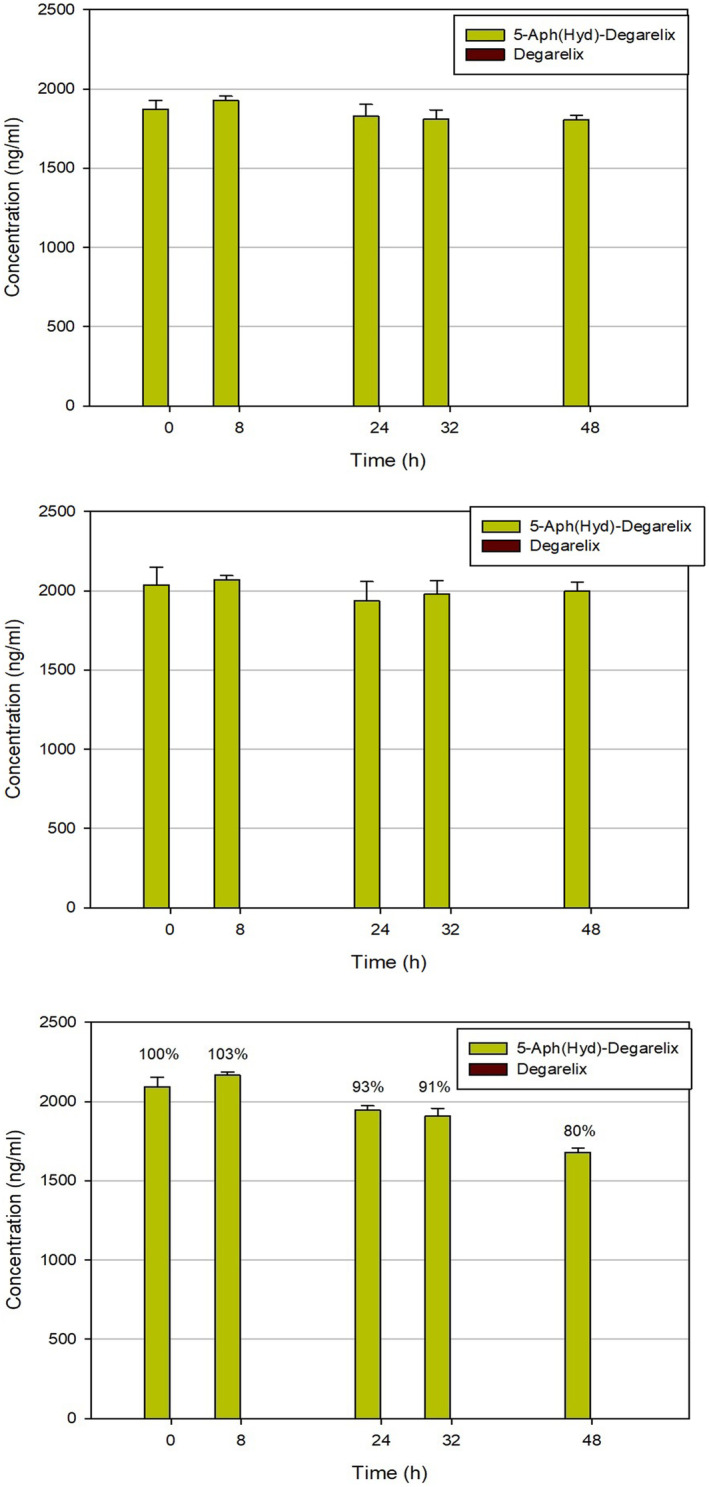
Stability of 5‐Aph(Hyd)‐degarelix (from top to bottom) in human serum albumin (HSA) solution without warfarin, in HSA solution with warfarin, and in human plasma.

To evaluate the reversibility of the dihydroorotate‐hydantoin isomerism, we also investigated the stability of 5‐Aph(Hyd)‐degarelix in the same testing environments (Figure 7). In human plasma, a gradual decrease of the 5‐Aph(Hyd)‐degarelix concentration was observed, which can be due to the degradation of the peptide or its aggregation in these conditions. No reverse formation of degarelix was detected over the period studied, confirming the irreversibility of the isomerism. On the contrary, in other matrices, we did not see any change in the 5‐Aph(Hyd)‐degarelix content.

## 4 DISCUSSION

Degarelix has emerged as a promising GnRH antagonist for prostate cancer treatment and its pharmacologic properties and metabolic pathways have been extensively studied during the past decade. The previous studies of degarelix metabolism in humans showed that the peptide is excreted unchanged via renal pathway, but it is sequentially degraded by the hepatobiliary system.^29^ Among the metabolites after 72 h mainly C‐terminally truncated nonapeptide FE200486 (1–9)‐OH was detected in plasma in the amount up to 6.3%.^29^ In vitro studies showed that degarelix was not a substrate for the human cytochrome P450 system and only very minor in vitro degradation was observed after incubation of the peptide in liver microsomes.^30^ However, in fresh hepatocytes, it was rapidly degraded to the nonapeptide metabolite. Thus, the origin of the nonapeptide in plasma samples could be due to the enzymatic degradation by endopeptidases located in the hepatic tissue.^29^ Metabolite pattern study allowed the detection of N‐terminal tetra‐ and penta‐peptides as main fragments formed during the passage of the hepatobiliary system.^31^

In previous studies of degarelix metabolism, chromatographic analysis of the samples was based on a gradient elution of the peptide by increasing the concentration of acetonitrile with 0.05% of trifluoroacetic acid. Here, we investigated the degradation of degarelix in various biological environments with the improved analytical method, which allows proper evaluation of the hydantoin isomer content. Unexpectedly, in human serum, we discovered a rapid formation of 5‐Aph(Hyd)‐degarelix when the experiments were carried out at 37°C. On the contrary, no hydantoin isomer was detected in the case of stability tests at low temperature. No hydantoin isomer was found in human serum albumin solution and HSA solution with the addition of the binding blocker warfarin. Thus, it can be confirmed the absence of the influence of albumin binding on this dihydroorotate isomerization. The kinetics of hydantoin formation showed that 5‐Aph(Hyd)‐degarelix was present already after 8 h. This finding indicates that the transformation of degarelix in vivo could be rather fast if compared with the rate of the release of the peptide from the depot to reach the maximum concentration in the blood (61.0–71.0 ng/mL in 37–42 h after starting dose injection) and leads to a decrease of the concentration of the circulating peptide.^32^ In this case, the contribution of the dihydroorotate‐hydantoin isomerization mediated by the slightly basic physiologic pH is negligible, as demonstrated by the experiments in phosphate buffer and human serum albumin solutions. Most probably, the previous studies of Degarelix degradation considered both the drug and its hydantoin metabolite as a single active substance, due to an inappropriate analytical method used. Indeed, the sum of these two compounds is close to the initial concentration of the drug when taking into account the formation of other metabolites, such as FE 200486(1–9)‐OH, which were not studied in this work.^29^ The study of the degradation of the corresponding hydantoin containing peptide did not show a reverse formation of degarelix in serum. The irreversible rearrangement of the dihydroorotate moiety of degarelix in human plasma at physiologic pH suggests that the isomerization is triggered by an enzymatic catalytic process. The possible mechanism could resemble those already proposed for the basic catalysis, which comprises the opening of the dihydroorotate ring, in this case potentially promoted by an enzyme soluble in plasma, for example, the CAD family of protein with dihydroorotase activity, followed by the subsequent closing to the thermodynamically favored hydantoin ring.^33^, ^34^ This pathway is partly justified by the observation that the 5‐Aph(Hyd)‐degarelix derivative does not isomerize back to degarelix in human plasma, due to the more favorable five‐member ring present in the hydantoin structure. An additional justification of this hypothesis can be the absence of the conversion at low temperatures where the metabolic processes are inhibited. However, the mechanism of this rearrangement and the enzyme(s) involved in the process require further investigation to fully elucidate the correct pathway. This study can be complicated by the low amount of the hydantoin metabolite. Indeed, the maximum concentration of the drug in the blood after starting dose for humans is about 60–70 ng/mL, which corresponds to about 8–9 ng/mL of the hydantoin isomer (13% based on our results). These values are out of range for the HPLC‐MS method proposed in this work. Thus, it cannot be suitable for in vivo studies without an additional concentration of the degarelix‐containing fractions. In vivo animal studies can be even more complicated when the starting dose is lowered with respect to that required for humans and the development of an alternative method for the quantification of the metabolite can be necessary.

The discovery of the rapid irreversible transformation of degarelix to hydantoin metabolite in serum needs a detailed study and suggests a critical re‐evaluation of the pharmacokinetics and pharmacodynamics of the drug, as well as the study of the potential activity of this metabolite as a GnRH antagonist. Moreover, this example highlights the requirement to develop more accurate and performing analytical techniques to adequately characterize the chemical composition of the manufactured drugs and their behavior in physiological conditions because of their increased structural complexity.

## AUTHOR CONTRIBUTIONS

Participated in research design: Antonio Ricci, Ulrich Abel, Marco Macis, Alessandra Tolomelli. Conducted experiments: Ulrich Abel. Contributed new reagents or analytic tools: Ulrich Abel. Performed data analysis: Ivan Guryanov, Antonio Ricci, Lucia Ferrazzano, Marco Macis. Wrote or contributed to the writing of the manuscript: Ivan Guryanov, Antonio Ricci, Walter Cabri

## CONFLICT OF INTEREST STATEMENT

The authors report no conflicts of interest.

## ETHICS STATEMENT

This article does not contain any studies with humans or animal participants. There are no human participants in this article and informed consent is not applicable.

## Data Availability

All data generated or analyzed during this study are included in this published article.
